# Ubiquitin Ligases at the Heart of Skeletal Muscle Atrophy Control

**DOI:** 10.3390/molecules26020407

**Published:** 2021-01-14

**Authors:** Dulce Peris-Moreno, Laura Cussonneau, Lydie Combaret, Cécile Polge, Daniel Taillandier

**Affiliations:** Unité de Nutrition Humaine (UNH), Institut National de Recherche pour l’Agriculture, l’Alimentation et l’Environnement (INRAE), Université Clermont Auvergne, F-63000 Clermont-Ferrand, France; dulce.peris-moreno@inrae.fr (D.P.-M.); laura.cussonneau@inrae.fr (L.C.); lydie.combaret@inrae.fr (L.C.); cecile.polge@inrae.fr (C.P.)

**Keywords:** skeletal muscle atrophy, hypertrophy, E3 ubiquitin ligase, MuRF1, MAFbx, anabolism, catabolism, signaling, therapy, treatment

## Abstract

Skeletal muscle loss is a detrimental side-effect of numerous chronic diseases that dramatically increases mortality and morbidity. The alteration of protein homeostasis is generally due to increased protein breakdown while, protein synthesis may also be down-regulated. The ubiquitin proteasome system (UPS) is a master regulator of skeletal muscle that impacts muscle contractile properties and metabolism through multiple levers like signaling pathways, contractile apparatus degradation, etc. Among the different actors of the UPS, the E3 ubiquitin ligases specifically target key proteins for either degradation or activity modulation, thus controlling both pro-anabolic or pro-catabolic factors. The atrogenes MuRF1/TRIM63 and MAFbx/Atrogin-1 encode for key E3 ligases that target contractile proteins and key actors of protein synthesis respectively. However, several other E3 ligases are involved upstream in the atrophy program, from signal transduction control to modulation of energy balance. Controlling E3 ligases activity is thus a tempting approach for preserving muscle mass. While indirect modulation of E3 ligases may prove beneficial in some situations of muscle atrophy, some drugs directly inhibiting their activity have started to appear. This review summarizes the main signaling pathways involved in muscle atrophy and the E3 ligases implicated, but also the molecules potentially usable for future therapies.

## 1. Introduction

Cachexia is a multifactorial syndrome leading to serious clinical complications with high mortality rates and is present in almost all chronic diseases [1]. Besides inflammation and metabolic modifications, skeletal muscle loss is an important factor of cachexia and limiting muscle wasting is a major challenge for maintaining well-being of patients, the capacity of the organism to fight against diseases and the tolerance of the patients towards challenging therapies like cancer chemotherapies [2].

Muscle homeostasis is mainly driven by the ubiquitin-proteasome system (UPS) that controls signaling pathways, contractile structure, cellular architecture, energy metabolism, protein translation, etc., thus allowing a fine-tuning of skeletal muscle metabolism [3,4,5,6]. The UPS is composed by hundreds of proteins and controls protein fate by ubiquitination, a post-translational modification carried out by the E1, E2, E3 enzymatic cascade (see [7] for a review). Ubiquitin (Ub) is covalently attached to the target proteins thanks to the interactions between Ub conjugating E2 enzymes (35–40 members according to species) and E3 Ub ligases (>600 in human). Another complexity of the UPS resides in the multitude of Ub signals that can be synthesized on the target proteins, from mono-Ub, multiple mono-Ub, or poly-Ub chains with at least eight different topologies. Each type of Ub modification is dedicated to a specific fate for the target protein, the role of some Ub linkages being still obscure. This Ub code can send the target protein for either proteasome or autophagy degradation or for non-proteolytic purposes (addressing, stabilization, activation, etc.) [7]. Furthermore, the multiple possible combinations between a given E3 and several E2s (and vice versa) further increase the potential of the UPS for controlling cellular metabolism.

E3 ligases can be either monomeric or multi-protein complexes and are classified into three families according to their structure and mode of action (recently reviewed [8]). The first class contains 28 members that contain a C-terminal Homologous to E6-Associated Protein C Terminus (HECT) domain that is necessary and sufficient to accept Ub from an E2 enzyme and to transfer it to the substrate, HECT E3 ligases having their own catalytic activity. Their N-terminal domain is involved in the recognition of the substrate. The second class comprises ≈90% of the E3 Ub ligases and are known as Really Interesting New Gene-finger (RING) type. RING domains are defined by eight cysteine and/or histidine residues coordinating four zinc atoms that allow interaction with E2 enzymes. RING-type E3s do not bind Ub, but they serve as a platform for the E2 and the substrate and promote the Ub transfer from the E2 to the substrate. Within multi-protein RING-E3 complexes, also named cullin-containing RING Ligase E3s (CRLs), several families of proteins with motifs involved in protein-protein interactions (e.g., F-box pattern) are responsible for substrate recognition [9]. The third class of E3 ubiquitin ligases are the RING-in-Between-RING (RBR)-type that combine properties of RING- and HECT-type E3s. They utilize an E2-binding RING domain and a second domain (called RING2) that binds Ub before transferring it to substrate [10,11].

Within muscle atrophy, numerous ubiquitinating enzymes are now identified for their involvement in the regulation of both anabolic and catabolic pathways during the atrophy process, notably by being responsible for the degradation of the contractile proteins [12]. The E3 Ub ligases appear to be at the heart of these regulations and some of them may prove to be efficient therapeutic drug strategies with roughly two main approaches: (i) indirect modulation of an E3 ligase by targeting the signals involved in its regulation [13,14,15,16] or (ii) direct inhibition of the E3 ligase [17,18,19]. However, the intertwinement between anabolic and catabolic processes (including the signaling pathways) often renders difficult an indirect modulation of E3 ligases, while direct inhibition strategies is limited by the somehow limited data available on E3 ligases.

This review summarizes the signaling pathways implicated in muscle homeostasis, and highlights the E3 ligases playing a role in the regulation of skeletal muscle mass and function, excluding the muscle regeneration process where numerous E3 Ub ligases are also involved. We more specifically focus on the strategies that have already been used for modulating E3 ligase activity, including pharmaceutical drugs or natural compound-based approaches.

## 2. Signaling Pathways Regulating Skeletal Muscle Mass and Function

**Figure 1:**
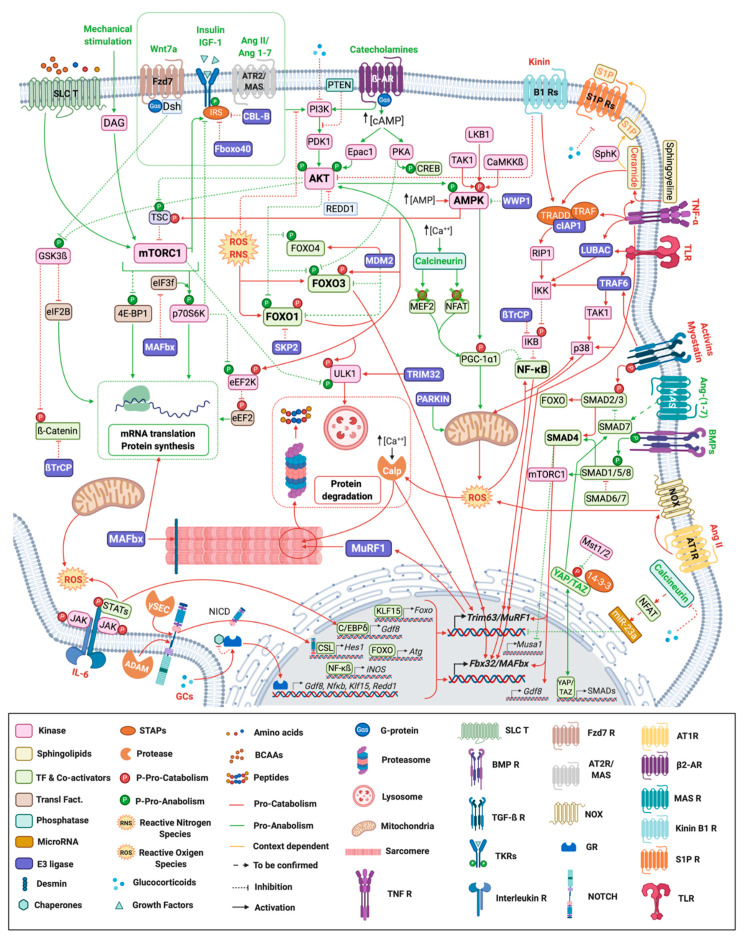
Signaling pathways regulating skeletal muscle mass and function. Myofiber representation of the different signaling pathways controlling skeletal muscle mass and function during atrophy conditions. Ligands and arrows (both with head or perpendicular line) in green denote those signaling pathways and interactions with an anabolic effect whereas the red ones represent catabolic signaling. Orange ligands and arrows stand for pathways with a dual role (context-dependent). ß2-AR: ß-2 Adrenergic Receptor; γ-sec: γ-secretase; Ang: Angiotensin; AT1R: Angiotensin II Type 1 Receptor; AT2R: Angiotensin II Type 2 Receptor; BCAAs: Branched-chain amino acids; BMP R: Bone Morphogenetic Receptor; Calp: Calpain; CSL: CBF1, Suppressor of Hairless, Lag-1; Dsh: Dishevelled; Fzd: Frizzled; GR: Glucocorticoid Receptor; IL-6: Interleukin-6; NCID: Notch Intracellular domain; NOX: NADPH oxidase activator; P: Phosphorylation; S1P: Sphingosine-1-phosphate; SLC T: Solute Carrier Transporter; STAPs: Signal Transducing Adaptor Proteins; TF: Transcription Factors; TGF-ß R: Transforming Growth Factor ß Receptor; TKR: Tyrosine-protein Kinase Receptor; TLR: Toll-like Receptor; TNF R: Tumor Necrosis Factor Receptor; Transl. Fact.: Translational Factors.

Skeletal muscle homeostasis is controlled by numerous signaling pathways (Figure 1) that act either as anabolic or catabolic factors. Depicting in detail their regulation is beyond the scope of this review and we just briefly summarize their implication in muscle mass control.

### 2.1. Anabolic Pathways

#### 2.1.1. PI3K/AKT Signaling Pathway

Skeletal muscle hypertrophy via the PI3K/AKT (phosphatidylinositol 3-kinase/protein kinase B) pathway can be induced by nutrients (amino acids, glucose and fatty acids) [20], hormones (insulin) [20,21] growth factors (Insulin Growth Factor-1 (IGF-1)) [22,23], and mechanical stimuli (e.g., exercise) [24]. Upon ligand binding, the PI3K/AKT pathway activates mTORC1 that phosphorylates numerous substrates [25,26], which regulate the activation of translation, transcription, ribosome biogenesis, and autophagy [27,28]. AKT also phosphorylates and inactivates GSK3β (a negative regulator of protein translation) [29] and the pro-catabolic FOXOs transcription factors (TF), the latter being crucial inducers of muscle loss upon catabolic situations via the expression of numerous atrophy-related genes [30,31,32,33]. Moreover, mTORC1 also inhibits the autophagy induction complex [34]. Intriguingly, mTORC1 can also exhibit adverse effects on skeletal muscle homeostasis upon denervation [35] or ageing [36,37]. In these situations, a negative feedback loop from mTORC1 to AKT was involved, thus favoring FOXOs activation and the subsequent expression of proteolytic genes like the atrophy-related E3 ligases *MuRF1/TRIM63* and *MAFbx/Atrogin-1*.

#### 2.1.2. G Protein-Coupled Receptors (GPCRs) and cAMP Signaling

1. ß2-Adrenergic Receptors Signaling Pathway

Upon stimulation by endogenous catecholamines or synthetic agonists, ß2-Adrenergic Receptors (ß2-ARs) lead to skeletal muscle hypertrophy (Figure 1) through: (i) PKA-mediated expression of genes containing cAMP response elements (follistatin, NR4A3, calpastatin) via CREB [38] (ii) PKA-mediated inhibition of FOXO activity in vivo [39] or (iii) the activation of PI3K/AKT/mTORC1 [40,41], or both AKT and CaMKII/HDAC4 signaling [42].

2. WNT/FZD Signaling Pathway

The Wingless-type mouse mammary tumor virus integration site (Wnt) family of proteins induce hypertrophy via Wnt/ß-catenin and PI3K/AKT/mTORC1 cascades [43,44] (Figure 1). The former one controls the transcriptional regulation of growth-related genes (e.g., *C-myc* and *Cyclin 1*) via ß-catenin and T-cell factor/lymphoid enhancer factor (TCF/LEF) transcription factors [45,46] whereas the latter regulates the protein synthesis process. The PI3K/AKT/mTORC1 pathway is induced via the specific interaction of WNT7a (ligand) and FZD7 (receptor) proteins [47,48,49,50]. Under mechanical stimulation, WNT is the only pathway able to stabilize ß-catenin and therefore to promote growth-related gene expression [51,52]. Accordingly, therapeutic stimulation of WNT7a/FZD7 by injection of recombinant Wnt7a resulted in a significant increase in muscle strength and a reduce contractile damages in mdx mice (Duchenne Muscular Dystrophy (DMD) model) [49]. By contrast, in dystrophic muscles WNT7a increased fibrosis by inducing transforming growth factor–β2 (TGFβ2) [53], and Wnt activation enhanced the fibrotic response in aged mice [54]. These data suggest WNT7a to have a context-dependent effect in skeletal muscle, thus complicating future therapeutic strategies.

#### 2.1.3. Calcineurin Signaling Pathway

Different downstream effectors have been proposed for calcineurin (Cn) during skeletal muscle hypertrophy, such as NFAT [55], GAT-2 [55] and MEF-2 [56], which seem to be activated during skeletal muscle hypertrophy in a fiber-specific manner [57]. Cn can modulate these TFs and downstream effectors (including the E3 ligases MuRF1/TRIM63 and MAFbx/atrogin-1) upon several conditions (dexamethasone [58], diabetes [56], exercise [59] or starvation [60] (Figure 1).

#### 2.1.4. Hippo Signaling Pathway

The Hippo signaling pathway consists of a cascade of kinases that inhibits the transcriptional co-activators YAP and TAZ (Figure 1) (for a review, see [61]). Upon exercise and myostatin/activin inhibition in *mdx* mice [62], mechanical overloading [63] and following injury or degeneration of motor nerves [64], the expression and phosphorylation of YAP increased [62,63] along with those of other pro-hypertrophy proteins [40]. Furthermore, YAP negatively regulated the myostatin/activins signaling pathway by inhibiting SMAD2/3 transduction and consequently blunted the SMAD-mediated MuRF1/TRIM63 E3-ligase expression [63].

### 2.2. Transforming Growth Factor (TGFs), Pro-Anabolic and Pro-Catabolic Pathways

The transforming growth factor (TGF) multifunctional cytokine family is divided in two subfamilies with opposite outcomes on muscle mass: myostatin/activin/TGF-β are negative regulators of muscle mass and BMPs (Bone Morphogenic Proteins)/GDF (Growth and Differentiation Factors) are positive regulators [65]. Myostatin/activin/TGF-β activate the pro-catabolic SMADs 2–3 whereas BMP ligands recruit pro-anabolic Smads 1-5-8 and elicit an anabolic transcriptional program (Figure 1). SMAD4 is shared by both pro-anabolic and pro-catabolic SMADs and can be a limiting factor for SMADs downstream effects [45].

Upon myostatin binding, Mafbx/Atrogin-1 and genes involved in the degradation of several anabolic factors (ribosomal proteins, translation initiation factors, MyoD, desmin and vimentin) are up-regulated [49,66] and the AKT/mTORC1 pathway is inhibited [67]. TGF-ß signaling also regulates *MuRF1/Trim63* expression through the synergistic action of FOXO3a and SMAD3 [68,69] (see [12] for a recent review). Similarly, Activin A ligand negatively regulates muscle mass by binding to the same receptor than myostatin and by activating the same intracellular pathway [70,71,72]. Interestingly, the non-canonical TGF-ß pathway involving TAK1-p38 MAP kinase can also be activated under Activin A treatment in cellulo and in vivo, with MAFbx-mediated myotube atrophy [73]. Moreover, TGF-ß induces skeletal muscle atrophy through a mechanism dependent on NOX-derived ROS production, in vivo [69]. The TGF-ß pathway is also known for its master role in fibrosis, which promotes muscle mechanical constraints and injuries [74,75]. Recent reports showed that the canonical NF-κB and angiotensin pathways mediate the TGF-ß effects in cellulo and in vivo [76].

Conversely, the BMP pathway regulates hypertrophy by repressing the E3 ligases MUSA1/Fbxo30 [77] MAFbx/Atrogin-1, MuRF1/Trim63 [78,79] and through the positive modulation of mTORC1 and consequently protein synthesis [80]. Additionally, the long non-coding RNAs Myoparr and Chronos negatively modulate the BMP pathway (and muscle mass) by repressing *Gdf5* [81] and *Bmp7* [82] respectively. Altogether, a major conceptual idea is that a net balance between TGF-ß/BMP pathways plays a major role in determining skeletal muscle mass.

### 2.3. Catabolic Pathways

#### 2.3.1. AMPK Signaling Pathway

The adenosine 5′-monophosphate-activated (AMP)-activated protein kinase (AMPK) is an energy sensor that preserves energy by turning on catabolic pathways and turning off ATP-consuming anabolic pathways [83,84,85]. In skeletal muscle, AMPK inhibits protein synthesis through the reduction of the mTORC1 signaling and favors contractile protein breakdown via the activation of FOXO1 and FOXO3a TFs (Figure 1) [86]. Consequently, MuRF1/TRIM63 and MAFbx/Atrogin-1 E3 ligases target different proteins involved in muscle contraction and protein synthesis initiation for UPS-dependent degradation [86,87]. Additionally, AMPK also promotes skeletal muscle autophagy [88].

#### 2.3.2. The NF-κB Signaling Pathway

**Figure 2:**
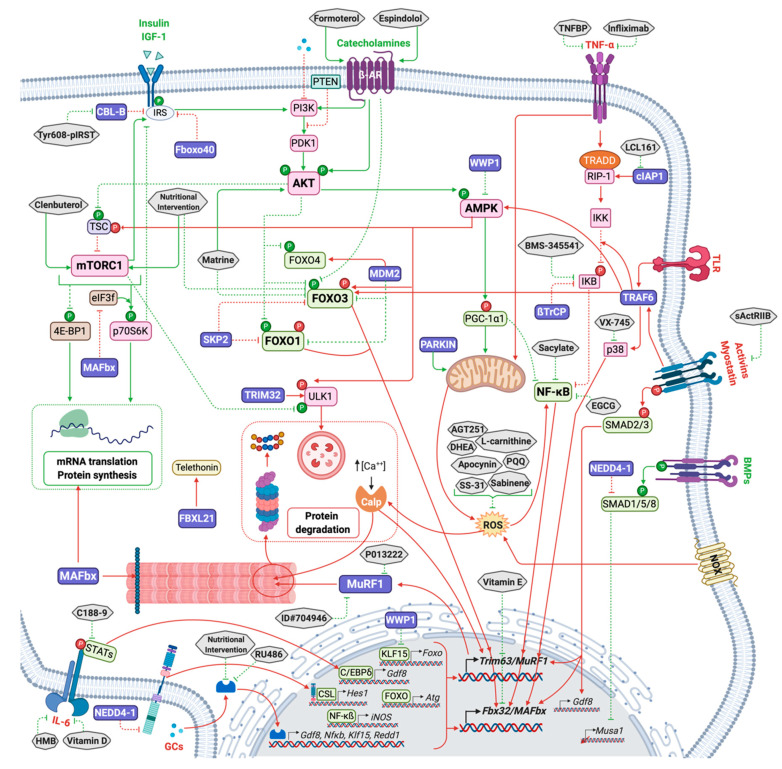

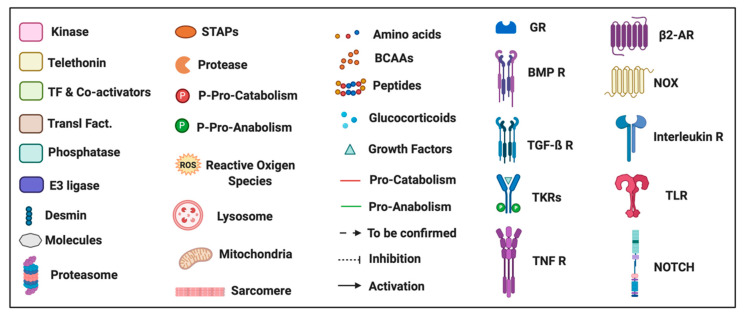
E3 ubiquitin ligases regulating skeletal muscle mass and molecules developed to modulate their activity and expression. Myofiber representation of the different E3-ligases and molecules targeting the signaling pathways controlling skeletal muscle mass and function during atrophy conditions. Ligands and arrows (both with head or perpendicular line) in green denote those signaling pathways and interactions with an anabolic effect whereas the red ones indicate catabolic signaling. ß2-AR: ß-2 Adrenergic Receptor; BCAAs: Branched-chain amino acids; BMP R: Bone Morphogenetic Receptor; Calp: Calpain; CSL: CBF1, Suppressor of Hairless, Lag-1; GR: Glucocorticoid Receptor; IL-6: Interleukin-6; NCID: Notch Intracellular domain; NOX: NADPH oxidase activator; P: Phosphorylation; STAPs: Signal Transducing Adaptor Proteins; TF: Transcription Factors; TGF-ß R: Transforming Growth Factor ß Receptor; TKR: Tyrosine-protein Kinase Receptor; TLR: Toll-like Receptor; TNF R: Tumor Necrosis Factor Receptor; Transl. Fact.: Translational Factors.

NF-κB, a major pro-inflammatory transcription factor, is considered one of the main effectors of muscle atrophy via the regulation of UPS-related proteins expression [89,90,91,92,93,94,95,96]. Indeed, the NF-κB pathway is consistently upregulated upon catabolic conditions in both mouse models [89,97,98] and patients suffering from chronic obstructive pulmonary disease (COPD) [99] or chronic heart failure (CHF) [100] patients. A hypertrophic response is also observed in myotubes when blunting NF-κB activation upon catabolic TNFα exposure [93]. In addition to TNFα induction of NF-κB signaling, other proinflammatory cytokines (such as IL6 and TWEAK), bacterial products, growth factors, ROS, genotoxic stress, and viruses can activate this pathway [101]. Interestingly, for controlling the proper signaling, the NF-κB pathway comprizes several E3 ubiquitin ligases, TRAF6 [95,102,103], cIAP1 [19,104], LUBAC [95,105], SCF^β-TRCP^ [105,106] that represent several opportunities for future potential therapies (Figure 2).

#### 2.3.3. Glucocorticoid Receptor Signaling Pathway

Glucocorticoids (GCs) are endogenous stress hormones involved in modulating inflammation [107]. GCs are well known for their catabolic effects on skeletal muscle [108] and can exert their action via different mechanisms (Figure 1). In skeletal muscles, GCs mainly operate through the glucocorticoid receptor (GR), that interacts with specific DNA sequences, DNA-bound TFs as well as transcriptional co-regulatory proteins, which modulate the transcription of numerous genes [108,109,110] like *MuRF1*/*Trim63*, *MAFbx/Atrogin-1*, *Foxo* transcription factors, the myokine *Gdf8*, *Klf15*, *Redd1* and *Sesn1* [110]. Intriguingly, the effect of GCs on muscle mass is dependent on the type of GC, fiber type composition, muscle type, sex and dose, but also on the type of catabolic situation (e.g., starvation, diabetes, sepsis, cancer cachexia, etc.) (for details, refer to [110,111]). Recent works at least partly explained these differential effects by the capacity of GCs to use different signaling pathways, such as IGF-1/PI3K/AKT, MEK/ERK, Myostatin [112], NF-κB [113], NOTCH [114] or to depend on co-factors such as connexin-based hemichannels [115], high-fat diet [116], oxidative stress [111] or mechanical load [51,52,117].

#### 2.3.4. Angiotensin Signaling Pathway

Angiotensin (Ang) is a peptide hormone that upon enzymatic processing [118] renders different variants like Ang-II and Ang-(1–7) (Figure 1) that can either be linked to catabolic conditions (Ang-II) [118,119,120,121,122,123] or counteract muscle atrophy (Ang-(1–7)) [124,125,126,127,128]. However, Ang-II can also exhibit anticatabolic properties, but only in some circumstances [127,129]). High levels of Ang-II have been associated with skeletal muscle atrophy in CHF, CKD, and SARS-CoV-2 pathologies [120,130]. Ang-II induced atrophy was also linked to increased proteasome activity [131], elevated polyubiquitinated protein conjugates [132], and early and transient accumulation of *MuRF1/Trim63* and *MAFbx/Atrogin-1* mRNA [119,123]. Therefore, the differential modulation of the enzymes processing Ang may be a promising approach for improving skeletal muscle atrophy.

#### 2.3.5. JAK/STAT Signaling Pathway

In skeletal muscle, the Janus Kinase/Signal Transducers and Activators of Transcription (JAK/STAT) pathway has been reported to be essential for transducing signals from growth factors and IL-6 among others (For a recent review, see [133,134]). STAT3, one of its effectors (Figure 1), is particularly implicated in skeletal muscle atrophy upon disease [recently reviewed elsewhere [135], notably through the development of skeletal muscle insulin resistance in Type 2 diabetes mellitus [136,137], the induction of myostatin [138], caspase-3 [139] and UPS [14], and increased mitochondrial ROS [140].

#### 2.3.6. Kinin Signaling Pathway

Kinins are a group of peptides that act via inducible (B1) or constitutive (B2) receptors [141]. Using B1 receptors, kinins participate to muscle atrophy by blunting the PI3K/AKT/mTORC1 axis and by stimulating the IKK/NF-κB pathway (Figure 1) [142]. Both genetic or pharmacologic ablation of B1 receptor protect skeletal muscles from atrophy in androgen-sensitive mice, mainly by blunting *MuRF1/Trim63* expression [142]. The role of kinin B2 receptors is more controversial as they may either be pro-catabolic via activation of myostatin signaling [143] or pro-anabolic [144]. Therefore, kinin receptors may regulate muscle mass but more studies are clearly needed before they become potential targets to modulate muscle atrophy.

#### 2.3.7. Sphingolipids Signaling Pathway

The sphingomyelin pathway plays a role in skeletal muscle mass through the hydrolysis of plasma membrane sphingomyelin (SM) and the subsequent formation of ceramide and sphingosine-1-phosphate (S1P) (Figure 1). Ceramide, is linked to muscle atrophy through (i) the reduction of protein synthesis [145,146,147,148] and (ii) the activation of NF-κB [149,150,151]. Oppositely, S1P can promote skeletal muscle mass in denervated mice [152] although the downstream signaling depends on the context and the S1P-receptor type [153].

#### 2.3.8. NOTCH Signaling Pathway

Hyperactivation of NOTCH leads to atrophy during cancer cachexia [154], denervation [155,156,157], chronic alcohol consumption [158], hypovitaminosis D [159], and glucocorticoid treatment [114]. Upon cleavage of the NOTCH receptor by secretases [160], the Notch Intracellular Domain (NICD) translocates to the nucleus (Figure 1) and binds directly to the *MuRF1/Trim63* promoter to activate its transcription, thereby establishing NOTCH signaling as a proteolysis inducer [161].

#### 2.3.9. Oxidative Stress Is an Inducer of Skeletal Muscle Atrophy

Oxidative stress is characterized by increased levels of reactive oxygen species (ROS) and/or reactive nitrogen species (RNS) and is a well-known mechanism of atrophy induction in skeletal muscle under several conditions and proteolytic mechanisms (reviewed elsewhere [162,163]) (Figure 1). Both ROS and RNS negatively impact muscle mass during COPD [164,165]. ROS induce a FOXO1-dependent *MuRF1/Trim63* and *MAFbx/Atrogin-1* overexpression in COPD peripheral muscle cells in cellulo [166]. NOS activation was suggested to occur through inflammation and hypoxia in COPD patients with low body weight via an activation of NF-κB and iNOS-generated RNS [99]. Besides increased protein breakdown, a decrease in protein synthesis via AKT/mTORC1 also contributes to muscle mass loss by ROS [162]. Importantly, depending on the type, duration and intensity of the imposed stress, specific signaling mechanisms are activated [162,163,166,167,168,169] indicating that the underlying mechanisms by which oxidative stress contributes to muscle wasting is context-dependent.

## 3. E3 Ligases Involved in the Regulation of Muscle Atrophy

### 3.1. E3 Ligases Involved in the Regulation of Anabolic Pathways

#### 3.1.1. The CBL-B and FBXO40 E3 Ubiquitin Ligases Target IRS1 to Degradation in Skeletal Muscle

One strategy to fight against atrophy may be to stimulate the anabolic pathways leading to skeletal muscle hypertrophy. Insulin-like growth factor 1 (IGF1) induces skeletal muscle hypertrophy by activating the IGF1R/PI3K/AKT pathway, a critical mediator and checkpoint being IRS1. Indeed, the effect of IGF1 is time-limited by the phosphorylation of IRS1 by IGF1R and its subsequent ubiquitination and proteasome-mediated degradation.

Different E3 ligases can target IRS1 in different tissues. For example, in embryonic fibroblasts, the CUL7 E3 ligase, containing FBXW8, has been shown to target IRS1 for ubiquitin-dependent degradation [170]. In skeletal muscle, Casitas B-lineage lymphoma-b (CBL-B), a RING E3 ligase, targets IRS1 for degradation and thus impairs muscular trophic signals in response to unloading conditions [171,172,173], which inhibits downstream IGF1 signaling [173] (Figure 2 and Table 1). Accordingly, mice deficient for CBL-B were partly resistant to unloading-induced skeletal muscle atrophy and dysfunction [173]. These results highlight the importance of CBL-B in the process of muscle atrophy in response to unloading.

FBXO40 is a muscle-specific F-box protein [174], component of an SCF (Skp1-Cullin1-F-box protein) E3 ligase complex. Following IRS1 activation, IGF1R phosphorylates IRS1 leading to its ubiquitination by FBXO40 and its degradation by the 26S proteasome, in cultured myotube and in mice [22,175]. FBXO40 expression is decreased in muscles from Limb-girdle muscular dystrophy (LGMD) patients, and up-regulated in mice skeletal muscle following denervation and in chronic kidney disease (CKD) mice model, but not during starvation [174,175]. Accordingly, the knock-down of *Fbxo40* resulted in thicker myotubes (20% to 50% increase in diameter) [22] and its deletion in mice also induced muscle hypertrophy during the growth phase, a phase associated with high IGF1 levels [22] (Figure 2 and Table 1).

IRS1 is thus an important checkpoint of the IGF1/PI3K/AKT pathway controlled by at least 2 E3 ligases (CBL-B and FBXO40). Although being an attractive target for fighting against muscle atrophy, the multiple ways for degrading IRS1 may complicate the development of drugs.

#### 3.1.2. NEDD4-1 E3 Ubiquitin Ligase, Friend or Foe?

In muscles undergoing atrophy, NEDD4-1 mRNA levels are elevated upon severe sepsis [191], denervation or unloading [178,192,193]. On the one hand, NEDD4-1 E3 Ub ligase targets phosphatase and tensin homologue (PTEN). PTEN is a redox sensitive phosphatase that negatively regulates the PI3K-AKT signaling pathway, thereby affecting metabolic and cell survival processes. The deletion of PTEN improves muscle mass and function in a mouse model of Duchenne muscular dystrophy [194]. PTEN inhibition may thus also represent a potential therapeutic strategy to maintain muscle function during catabolic situations. The over-expression of NEDD4-1 is sufficient for activating the PI3K/AKT signaling in cardiac muscle, following myocardial ischemia/reperfusion (I/R) [176]. However, the negative regulation of PTEN by NEDD4-1 remains to be confirmed in skeletal muscle, especially since NEDD4-1 has also been shown to promote skeletal muscle atrophy in a denervation model. Indeed, NEDD4-1-KO mice exhibited increased weights and type II muscle fiber cross-sectional areas in denervated gastrocnemius muscle [178]. Moreover, NEDD4-1 also negatively regulates the hypertrophic BMP signaling (Figure 1 and Figure 2). Indeed, NEDD4-1 ubiquitinates phosphorylated-SMAD1 leading to its proteasomal degradation, thereby silencing BMP signaling in C2C12 myoblasts, and conversely the knock-down of *Nedd4-1* potentiates BMP signal through upregulation of phospho-SMAD1 [195]. Altogether, the exact function of NEDD4-1 in skeletal muscle is still obscure and needs more work.

### 3.2. E3 Ubiquitin Ligases Involved in the Regulation of Catabolic Pathways

#### 3.2.1. Regulating the Canonical NF-κB Pathway via the Manipulation of cIAP and TRAF6 E3 Ligases

Among the E3s involved in the regulation of the NF-κB pathway, two promising candidates may be manipulated to limit muscle atrophy, namely cIAP and TRAF6 (Figure 1 and Figure 2). cIAP1 is up-regulated in denervated gastrocnemius muscle, paralleling the upregulation of *MAFbx/atrogin-1* and *MuRF1/Trim63* mRNA [19]. Mice with genetic ablation of cIAP1 (cIAP1-KO mice) displayed limited denervation-induced atrophy in TA, gastrocnemius and EDL muscles. This was correlated with the blunting of the denervation-induced upregulation of *MAFbx/Atrogin-1* and *MuRF1/Trim63* [19]. The authors further demonstrated that cIAP1 induced atrophy through the up-regulation of the canonical NF-κB signaling. Conversely, cIAP1 overexpression in myotubes induced atrophy and the strong up-regulation of *MAFbx/Atrogin-1* and *MuRF1/Trim63* protein expression [19]. The E3 Ub ligase cIAP1 represents thus a potential therapeutic target at least for fighting against denervation-induced muscle atrophy.

TRAF6 is a RING-type Ub ligase that plays an important role during skeletal muscle atrophy. TRAF6 expression is enhanced during starvation or within aged-induced muscle atrophy [179,196,197]. *Traf6*-KO mice are resistant to skeletal muscle loss (rescue of myofibril degradation, preservation of myofiber size and strength) induced by denervation, cancer cachexia, starvation or Dex and a concomitant suppression of the expression of key regulators of muscle atrophy was observed, including MAFBx/Atrogin-1, MuRF1/TRIM63, p62, Lc3b, Beclin1, Atg12, and Fn14 [179,180,196,197,198]. Moreover, inhibition of *Traf6* expression through miR-351 administration in C2C12 myotubes or in denervated mice attenuated Dex-induced muscle atrophy and concomitantly decreased the expression of *MAFBx/Atrogin-1* and *MuRF1/Trim63* [199,200]. Overexpression of miR-125b targeted *Traf6* for degradation and protected skeletal muscle samples from atrophy in starved myotubes or in denervated rat tibialis muscle [201]. The implicated mechanisms involved both direct and indirect effects of TRAF6 on protein breakdown with TRAF6-mediated ubiquitination being required for the optimal activation of JNK, AMPK, FOXO3, and NF-κB catabolic pathway in muscle [202].

In human, gastric cancer patients suffering from cachexia exhibited an upregulation of TRAF6 associated with an upregulation of ubiquitination in the rectus abdominis muscle [203]. Altogether, this highlights the importance for targeting TRAF6 inhibition to counteract muscle atrophy.

#### 3.2.2. WWP1 in the Regulation of Muscle Atrophy

WWP1 is a HECT E3 ligase that is involved in chicken muscular dystrophy. Indeed, a missense mutation in the gene coding WWP1 was identified as the most promising candidate responsible for chicken muscular dystrophy (MD), potentially affecting the E3 function of WWP1 protein [204]. WWP1 was also shown to target the transcription factor KLF15 [181]. In response to glucocorticoids, KLF15 is up-regulated at the mRNA levels [205]. This induction leads to the up-regulation of the E3 ligases *MAFbx/Atrogin-1* and *MuRF1/Trim63* expression, likely in cooperation with a FOXO transcription factor, while inhibiting the anabolic mTORC1 [205]. Likewise, exogenous KLF15 expression in myotubes and in TA muscle leads to myofiber atrophy [205]. It has recently been shown that KLF15 protein expression was upregulated in skeletal muscle of diabetic mice, without any change in its mRNA expression [181]. This increase correlated with an increase in *MAFbx/Atrogin-1*, *Murf1/Trim63* and *Foxo3* genes expression and accordingly, the muscle-specific deletion of *Klf15* in this model prevented from diabetes-induced muscle atrophy [181]. The authors identified WWP1 as an E3 ligase targeting KLF15 and showed that knocking-down WWP1 in both C2C12 myotubes and in tibialis anterior muscles increased *MuRF1/Trim63* and *MAFbx/Atrogin-1* expression and induced atrophy [181] (Figure 2). WWP1 E3 ligase is indeed induced by high glucose conditions in myotubes [206]. Conversely, in high glucose conditions, WWP1 has also been implicated in the down-regulation of AMPKα2 protein levels [206]. The authors have shown that WWP1 interacted with AMPKα2 leading to a proteasome-dependent decrease of AMPKα2 in myotubes; however, direct ubiquitination was not addressed [206]. WWP1 may thus control muscle mass through a direct action on AMPK, a known modulator of FOXO3a, MuRF1/TRIM63 and MAFbx/Atrogin-1 [88].

#### 3.2.3. TRIM32 in the Regulation of Autophagy

TRIM32 is a RING E3 Ub ligase whose mutation is responsible for the development of limb girdle dystrophy 2H (LGMD2H) [207]. Several substrates have been identified for TRIM32 in non-muscle cells, including cell cycle regulators (c-Myc, MYCN, p53), the cell growth and transformation factor ABI2 and PIASY (a SUMO E3 ligase). TRIM32 is also involved in the targeting of factors influencing myogenesis (NDRG2 and TRIM72) that regulate muscle satellite cells renewal and differentiation [208]. While initially postulated to promote muscle atrophy, TRIM32 is in fact a master regulator of myogenesis during recovery situations [208]. Indeed, the dystrophic phenotype of TRIM32 mutations appeared to be largely due to impaired myogenesis [208,209,210].

More recently, TRIM32 was implicated in the early events leading to autophagy. Indeed, TRIM32 targets ULK1, a Ser/Thr protein kinase (Figure 1 and Figure 2). ULK1 is an upstream regulator of autophagy rapidly activated to ensure a rapid response to stress conditions [211]. The authors showed that TRIM32 deficiency was directly responsible for autophagy defects both in cultured cells and in mice treated with Dex. The mechanisms by which TRIM32 controls the activation of autophagy through ULK1 involves its binding to AMBRA1, a positive regulator of autophagy [211]. AMBRA1 is a pivotal factor able to bind several E3 ligases during the course of the autophagy process. In presence of AMBRA1, TRIM32 binds to ULK1, synthesizes unanchored K63 Ub chains that activate ULK1 kinase activity, thus promoting autophagy. The role of TRIM32 during the autophagy process is not limited to ULK1 as p62, an important autophagy receptor [212], is also a TRIM32 substrate. p62 activity is modulated by multi mono-Ub catalyzed by TRIM32 and loss of function of TRIM32 largely abolished autophagy [213]. Altogether, TRIM32 appears as a master regulator of muscle renewal through the initiation of autophagy.

#### 3.2.4. FOXO Transcription Factors Are Regulated by MDM2 and SKP2 E3 Ubiquitin Ligases

Alternatively to phosphorylation, FOXO can be regulated by acetylation/deacetylation, methylation and ubiquitination to modulate its activity, localization as well as degradation [214,215,216].

Ubiquitination modulate FOXO activity by either mono- or polyubiquitination through MDM2 and SKP2 E3 Ub ligases (Figure 1 and Figure 2). MDM2 is the enzyme responsible of a single addition of an ubiquitin moiety to FOXOs, specifically to FOXO4, thus allowing its nuclear localization and transcriptional activation [217,218]. Mono-Ub of FOXO4 is observed under oxidative stress conditions and can be counteracted by deubiquitinating enzymes such as ubiquitin-specific protease (USP7). Importantly, ubiquitination mediated by MDM2 is context specific and upon growth factor stimulation can induce FOXO1 and 3 degradation [217]. In addition, interaction between FOXOs and SKP2, a subunit of the SKP/cullin 1/F-box protein E3 ligase leads to proteasomal degradation of FOXO1 in the cytosol [218].

Combined with the other posttranslational modifications, ubiquitination allows FOXOs to integrate information arising from insulin, growth factors, cytokines, and oxidative stress and to control downstream signaling. Interestingly, FOXO TFs have systematically been envisioned as crucial drivers of catabolic pathways during muscle wasting. Nonetheless, recent work showed that FOXO1 and 3a participate to skeletal muscle adaptation upon exercise thus adding a new of FOXOs in the control of muscle cell homeostasis [219,220,221,222].

### 3.3. E3 Ubiquitin Ligases Involved in the Regulation of Muscle Mass and Function

#### 3.3.1. MuRF1/TRIM63

Muscle-specific RING finger protein 1 (MuRF1), also named TRIM63, is a RING-type E3 ligase and a founding member of the so-called “atrogenes” (see [6] for a recent review). MuRF1/TRIM63 is a master regulator of skeletal muscle atrophy development occurring in numerous catabolic conditions and *MuRF1/Trim63* mRNA appeared to be upregulated in more than 25 atrophying situations [6] (Figure 1 and Figure 2). Mice deleted for MuRF1/TRIM63 (MuRF1-KO mice) were partially resistant (preservation of muscle mass and structure) to skeletal muscle atrophy induced by denervation [4], hindlimb suspension [4,223], glucocorticoid [224], amino acid deprivation [225], and acute lung injury [226]. MuRF1/TRIM63 is responsible for the coordinated breakdown of both thick and thin filaments occurring during catabolic states in skeletal muscle, targeting to degradation the main proteins of the contractile apparatus: myosin heavy chains (MHC) [227], alpha-actin [228], troponin I [229], TCAP/telethonin [230]. During denervation and starvation, MuRF1/TRIM63 has also been involved in the degradation of acetylcholine receptor (CHRN), the major postsynaptic ion channel of the neuromuscular junction. This degradation is mediated by the activation of selective autophagy and degradation of CHRN, likely via the degradation of BIF-1 (Bax interacting factor 1)/EndoB1 (EndophilinB1) and/or SQTM1/p62 (sequestosome-1) [231,232].

While numerous studies have promoted a major role of MuRF1/TRIM63 in the development of skeletal muscle atrophy during catabolic states, in the heart, the analyses of MuRF1 mutants have highlighted a beneficial cardioprotective role [233]. These opposites roles in both kind of muscles imply the development of skeletal muscle-specific drugs to inhibit MuRF1/TRIM63. Moreover, one should also take into account that MuRF1/TRIM63 has two homologs, MuRF2 and MuRF3 that share some redundant functions and could replace its role [12].

#### 3.3.2. MAFbx/Atrogin-1/FBXO32

The multimeric E3 ligase MAFbx/atrogin-1/FBXO32 is another founding member of the atrogene family ([6] for a recent review) crucial for the development of muscle atrophy. Interestingly, nearly all catabolic situations induce an overexpression of both MAFbx/Atrogin-1 and MuRF1/TRIM63, which are controlled by the same TFs (FOXO1/FOXO3a, NF-κB, C/EBP β, Smad 3, etc.) and the same signaling pathways [234] (Figure 1 and Figure 2).

In contrast with MuRF1/TRIM63 that targets directly the contractile proteins for their degradation (α-actin, MHC, etc. [227,228,229,230], MAFbx appeared to target pro-anabolic factors like MyoD, myogenin or eIF3f [235,236,237]. MyoD is a muscle-specific transcription factor that plays crucial roles during cell cycle and muscle differentiation [238]. The eukaryotic initiation factor 3 subunit f (eIF3f) is a pivotal element of protein synthesis and its control by MAFbx allows the latter to master the anabolic processes [235]. While a putative role of MAFbx/Atrogin-1 on sarcomeric proteins was hypothesized using an indirect approach, this has never been confirmed [239]. By contrast, the authors found that desmin, a main component of the intermediate filaments, physically interacted with MAFbx and was degraded in myostatin-treated cultured C2C12 myotubes.

As MAFbx/Atrogin-1 and MuRF1/TRIM63 are controlled by similar signaling pathways, the strategies for the upstream control of *MuRF1/Trim63* expression are generally also valid for MAFbx/Atrogin-1 (Table 2). By contrast with MuRF1/TRIM63, no direct inhibitor of MAFbx/Atrogin-1 has been described so far but general strategies, like targeting the interface responsible for substrate recognition or impeding the assembly of the F-box (i.e., the subunit recognizing the substrates) into the SCF complex, may prove to be efficient.

Altogether, controlling concomitantly MAFbx/Atrogin-1 and MuRF1/TRIM63 E3 ligases allows skeletal muscle cells to both increase the degradation of the contractile apparatus and to depress the protein synthesis machinery, which allows a tight regulation of protein homeostasis.

#### 3.3.3. PARKIN Controls Muscle Mass through the Maintenance of Mitochondrial Homeostasis

PARKIN is an E3 ubiquitin ligase implicated in the regulation of mitophagy, a quality control process in which defective mitochondria are degraded. Mitochondrial quality control through both mitochondria turnover and dynamic plays an essential role in the maintenance of muscle mass (see [240] for a review). During mitophagy, PARKIN ubiquitinates several outer mitochondrial membrane proteins leading to subsequent autophagosomal engulfment and lysosomal degradation (Figure 1 and Figure 2).

This role of PARKIN has been emphasized in rodent models or in humans where a deregulation of PARKIN mRNA and/or protein expression prevailed in response to catabolic or anabolic situations. An accumulation of PARKIN protein prevailed during: (i) muscle wasting situations such as chronic kidney disease [241], chronic obstructive pulmonary disease (COPD) [242], physical inactivity [243,244] and (ii) upon exercise training [245,246]. Conversely, PARKIN mRNA or protein levels decreases in skeletal muscles from some elderly populations, perhaps related to the loss of muscle mass and poor physical function, e.g., physically inactive frail older women [247,248] or gastric cancer patients with cachexia [249].

In the last two years many studies using loss/gain of function models have provided insight on the role of PARKIN in skeletal muscle. Loss of function mouse models pointed out the essential role of PARKIN in basal conditions for the maintenance of (i) mitochondrial function [250,251] and (ii) skeletal muscle mass and normal contractile function [184,251]. Such studies also reported that PARKIN helps to resist to some drug-induced muscle damages [252] and is required for exercise-induced mitophagy flux and for the accumulation of functional mitochondria following muscle adaptations to training [250]. In addition, these loss-of-function studies also highlighted that PARKIN-mediated mitochondrial clearance contributes to proteasome activation during denervation in atrophied slow-twitch muscles [253]. On the flip side, gain-of-function studies showed that PARKIN overexpression in mice: (i) attenuates the ageing-related and the sepsis-induced muscle wasting and causes hypertrophy in adult skeletal muscle, (ii) increases mitochondrial content and enzymatic activities and (iii) protects from ageing-related increases of oxidative stress markers, fibrosis and apoptosis [185,186]. It is very likely that this role of PARKIN in controlling muscle mass has been evolutionary conserved. Indeed, similar observations were also reported in the fruit fly model: *Parkin* deficiency in *Drosophila* leads to severe degeneration of the flight muscles with accumulation of swollen mitochondria [254] whereas *Parkin* overexpression promotes mitophagy in older muscles and extend lifespan.

Together, these studies clearly indicate that PARKIN is an important player in the control of muscle mass through its role in the maintenance of mitochondrial homeostasis. This makes it a potential therapeutic target of interest for preserving muscle mass or fighting against atrophy. Nevertheless, the regulation of PARKIN can be very different according to the physiological or pathological situation or during ageing. Further investigations should enable defining how this actor could be a target of interest according to the population considered.

#### 3.3.4. MUSA1/FBXO30

FBXO30, also called muscle ubiquitin ligase of the SCF complex in atrophy-1 (MUSA1), is a FBOX protein forming an SCF complex with SKP1, Cullin1 and ROC1 [77]. Proteins targeted by MUSA1 remain undefined, but its inhibition in denervated muscles reduces remarkably muscle atrophy, and reverts almost completely the strong atrophic phenotype of *Smad4*-KO mice [77] (Figure 1 and Figure 2). In muscle, *Musa1* expression is upregulated in atrophic mice muscle undergoing CKD [255] or sepsis [256].

#### 3.3.5. FBXL21

Very recently, a new E3 ubiquitin ligase involved in muscle function control has emerged, FBXL21 [188]. FBXL21 forms an SCF E3 ligase complex and was first identified as clock-controlled E3 ligase modulating circadian periodicity via subcellular cryptochrome degradation [257]. Accordingly, in mice, the *Psttm* mutation, corresponding to a hypomorphic mutation of FBXL21 with reduced FBXL21 activity, caused circadian period shortening [257]. Further studies of these mice revealed that they also displayed skeletal muscle deficiencies with a decrease in fiber CSA (gastrocnemius) and impaired exercise tolerance and grip strength for both forelimbs and hindlimbs [188]. The authors nicely demonstrated the circadian degradation of the cytosolic TCAP/Telethonin by FBXL21 (Figure 2), under the control of GSK-3β. They reported that GSK-3β phosphorylated both FBXL21 and TCAP leading to FBXL21-CULLIN1 complex formation and phosphodegron-dependent TCAP turnover.

#### 3.3.6. Ubiquitin Ring-Type E3 Ligases (UBR)

Ubiquitin Ring-type (UBR, also referred to as E3α) proteins are RING finger E3 ligases that compose a 7-member family and that mainly recognize their substrate through the N-end rule pathway [258]. A first member, UBR2/E3alpha-II, has been shown to be significantly induced in skeletal muscle, in two different animal models of cancer cachexia, at the onset and during the progression of muscle wasting [259]. However, its exact function and importance in skeletal muscle maintain during catabolic states have not been further studied. UBR4 is overexpressed in the skeletal from fasted mice and genetic ablation of UBR4 preserves muscle mass in tumor-bearing mice [189] (Table 1). Intriguingly, the protection of UBR4 knockout against tumor-induced atrophy was limited to type IIA fibers. In contrast, UBR5 has been implicated in muscle hypertrophy [260] and reported to be at least partially associated to the proteasome [261]. Recently several members of the UPS have been described as UBR5 substrates, which included an E2 (UBE2B, an abundant muscle E2), several E3 ligases, proteins involved in chromatin remodeling, etc. [189]. As the main UBR4 targets are positive regulators of muscle growth, the authors concluded that UBR4 acts as a negative regulator of muscle hypertrophy.

#### 3.3.7. FBXO21/SMART

FBXO21/SMART forms an SCF complex with Skp1, Cullin1 and Roc1, in skeletal muscle and has been shown to promote atrophy during denervation [187]. Indeed, the authors showed that FBXO21/SMART upregulation was required for atrophy while, knock-down in TA muscle protected denervated muscles from atrophy (Table 1), probably due to a global reduction of protein ubiquitination [187]. FBXO21/SMART might therefore be a new critical E3 to target to limit skeletal muscle atrophy. Further work should determine whether this E3 is crucial for the development of atrophy in other catabolic conditions and what are the mechanisms involved.

### 3.4. Promising E3 Ubiquitin Ligases Regulating Muscle Mass and Function

Other E3 ubiquitin ligases are also promising putative targets for maintaining muscle mass and function, if we rely on what has been published in other organs or organisms. For example, the SIAH-1 RING E3 ligase has been identified in the same RNAi screen that UBR4, performed to identify ubiquitin-related enzymes that regulate myofiber size, using the fruit fly *Drosophila* [189]. In *Drosophila*, SIAH1 knock-down led to muscular hypertrophy while its overexpression led to atrophy [189]. It is noteworthy that, in space flown rats, *SIAH1* mRNA expression has been shown upregulated suggesting also a putative role during this process in mammals [172]. However, in mammals two isoforms, SIAH1 and SIAH2, are expressed in muscle and could share redundant functions [189].

SMURF1, an HECT ubiquitin ligase interacts with SMAD1 and SMAD5 (BMP pathway) and SMAD4 in a certain context, leading them all to proteasomal degradation in vitro [262]. Moreover, it can degrade the main TGF-β receptor through an indirect recruitment to the receptor by SMAD7, leading to the receptor degradation [263]. In, COPD leading to muscle atrophy, TGF-β signaling is abnormally up-regulated and this, is negatively correlated to SMURF1 expression. This highlights that the inhibitory effect of SMURF1 over TGF-β is needed for muscle homeostasis [264].

The C terminus of Hsc70-interacting protein (STUB1/CHIP) serves as an E3 ubiquitin ligase. This E3 plays a dual role in BMP/TGF signaling. Overexpression of CHIP inhibits TGF-β luciferase reporter through the ubiquitination and degradation of SMAD3, and conversely silencing it leads to increase the signal transduction in HEK293T cells [265]. In cellulo experiments showed that CHIP mediates as well SMAD1-5 poly-ubiquitination, and subsequent degradation to terminate BMP signaling [266]. In muscle, CHIP is highly expressed. For instance, *Chip*−/− mice at 6 months shows muscle morphological changes consistent with increased sarcoplasmic reticulum compartments in quadriceps muscle and gastrocnemius, resulting in damages and fiber switch composition [267]. From our knowledge, no studies have shown the implication of CHIP in TGF/BMP signaling-mediated muscle atrophy.

TRIM62 belongs to the TRIM/RBCC family. This enzyme acts as a negative regulator of TGF-β signaling by binding to SMAD3 and promoting its ubiquitination and degradation, resulting in a decrease of TGF-β/SMAD3 target genes in HEK and human mammary epithelial cells [268]. TRIM62 is increased in the skeletal muscle of ICUAW patients (Intensive care unit-acquired weakness), a devastating illness characterized by loss of muscle mass [269]. In this context, the authors proposed TRIM62 contribution in inflammation-induced muscle atrophy through IL-6 pathway. Indeed, *Trim62-KD* inhibited LPS-induced IL-6 expression in C2C12 cells [269].

TRIM72/MG53 is a muscle-specific E3 ligase, also called mitsugumin 53, specifically expressed in the plasma membrane of skeletal muscle, and has a critical role in membrane repair. Membrane repair deficiency causes muscle cell death, injury, and dystrophy. Accordingly, the overexpression of human TRIM72 in a hamster model of genetic muscular dystrophy protects skeletal muscle damage through enhancement of membrane repair [270]. Similarly, short-term TRIM72 injection ameliorates the underlying defects in dysferlin-deficient muscle by increasing sarcolemma membrane integrity [271] while *Trim72*^−/−^ mice develop significant skeletal muscle myopathy and cardiovascular defects due to defective sarcolemma repair [272].

## 4. Current Treatments/Potential Modes of Action

The importance of maintaining muscle mass together with the discovery of several E3 ligases implicated in muscle homeostasis has rapidly end up with multiple approaches to chemically alter the expression of these enzymes. This includes chemical drugs but also several natural molecules that have been tested for their ability to modulate the UPS and more particularly the E3 ligases (Table 2).

### 4.1. Indirect Action on E3 Ligases

#### 4.1.1. PI3K-AKT-mTORC1

As E3 ligases are controlled by several signaling pathways, one possibility that was first addressed was to block these signals. The PI3K-AKT-mTORC1 axis is known to control muscle mass by directly acting on FOXO transcription factors, the latter being master regulators of several E3 ligases, like MAFbx/Atrogin-1, MuRF1/TRIM63, MUSA1, SMART and FBXO31, during several atrophy situations [187]. As such, clenbuterol (Table 2 and Figure 2), an activator of the AKT-mTORC1 pathway, is able to decrease *MuRF1/Trim63* and *MAFbx/Atrogin-1* expression in denervated or hindlimb suspend rats and to partially preserve muscle mass [273].

#### 4.1.2. Glucocorticoids

Glucocorticoids are potent manipulators of muscle mass and the glucocorticoid receptor antagonist RU486 proved to be efficient in rats for blocking dexamethasone (Dex)-induced induction of *MuRF1/Trim63* of *MAFbx/Atrogin-1*, the main regulators of muscle mass [13] (Table 2 and Figure 2). Similarly, the authors demonstrated that blocking TNFα by the TNF-binding protein (TNFBP) was efficient for blunting LPS-induced expression of *MuRF1/Trim63* and *MAFbx/Atrogin-1*. However, when sepsis was induced by cecal ligation and puncture, neither RU486 nor TNFBP were able to counteract the overexpression of *MuRF1/Trim63* and *MAFbx/Atrogin-1*, indicating that multiple signals were activated by sepsis. This points out the difficulty of treating complex catabolic signals in vivo. Infliximab is an anti- TNF-α agent able to lower the downstream NF-κB signaling. In patient’s suffering from Crohn disease, treatment with infliximab was able to ameliorate muscle atrophy but, although hypothesized by the authors, the expression of *MuRF1/Trim63* or any other E3 ligase was not addressed [274].

#### 4.1.3. Il-6

Il-6 is another inflammatory cytokine that can be implicated during muscle wasting conditions like muscle disuse [275]. Increased IL-6 in tail-suspended mice paralleled skeletal muscle atrophy and was accompanied by increased levels of *MuRF1/Trim63* and *MAFbx/Atrogin-1*. The inhibition of the IL-6 receptor by hydroxymethyl butyrate (HMB, a metabolite of leucine) or vitamin D tended to decrease IL-6 levels and when combined, HMB and vitamin D exhibited better efficiency for blunting IL-6 production [275] (Table 2 and Figure 2). By contrast, each molecule was sufficient for decreasing MuRF1/TRIM63 and MAFbx/atrogin-1 levels and to attenuate muscle atrophy. While the authors attributed the beneficial effects of HMB and vitamin D on IL-6 receptor, using a monoclonal antibody directed against IL-6 receptor (MR16-1) proved to be inefficient as only *MuRF1/Trim63* expression was decreased with no amelioration on muscle mass. As for the TNF-α, this work underscores the multiplicity of signaling during atrophy situations and the difficulty of blunting efficiently receptor-linked signaling. STAT-3 is a downstream effector of IL-6 signaling and a specific inhibitor of STAT-3 (C188-9) was investigated for its capacity to block muscle atrophy in a model of mice deficient for the vitamin D receptor (VDR) [14]. In these conditions, VDR^−/−^ mice exhibited exacerbated *MuRF1/Trim63* expression and increased muscle atrophy. While C188-9 was able to partially preserve muscle mass, its efficacy against MuRF1/TRIM63 was not addressed.

#### 4.1.4. NF-κB

Inhibition of the NF-κB signaling pathway was also efficiently performed using high doses of salicylate (Table 2 and Figure 2), which allowed the reversion of MuRF1-induced muscle atrophy in tumor bearing or denervated mice [89]. However, the high doses used for achieving a potent inhibitor would be toxic when administered to humans.

#### 4.1.5. ß2 Adrenergic Receptor (β2-AR)

β2-AR agonists can exert both anabolic and anti-catabolic effects on skeletal muscles either by decreasing catabolic signals or by promoting anabolic ones or both. Formoterol (Table 2 and Figure 2), a β2-AR agonist, was shown to reverse *MuRF1/Trim63* and *MAFbx/Atrogin-1* overexpression with a concomitant muscle sparing in tumor-bearing mice [276]. Intriguingly, neither a repression of FOXO1 and FOXO3a transcription factors nor an activation of AKT-mTORC1 pathway explained the positive effect of formoterol. By contrast, formoterol was able to blunt *MuRF1/Trim63* and *MAFbx/Atrogin-1* expression in LPS-induced muscle atrophy through restoration of the AKT-mTORC1 pathway and reversal of P-FOXO/FOXO1 ratio [277].

β2-AR reversion of E3 ligases expression and muscle sparing was also observed in a rat rheumatoid arthritis model and was attributed to modulation of both the AKT and the NF-κB pathways [293]. Other 2-AR agonists like espindolol have also been shown to ameliorate muscle loss and to blunt E3 ligase expression in aged rats. The authors found that both NF-κB and myostatin expression was reduced with no effect on AKT and FOXO3a [292]. Altogether, this strongly suggests that the positive effects of 2-AR agonists on muscle mass are mediated through the modulation of different signaling pathways depending on the catabolic stimuli, which complicates future therapeutical strategies.

#### 4.1.6. p38α Mitogen-Activated Protein Kinase (p38α MAPK)

p38α MAPK is known to play an important role in the development of muscle atrophy [295]. Inhibition of the p38α MAPK receptor by the selective inhibitor VX-745 (Table 2 and Figure 2) partially improved muscle weight in hindlimb suspended rats with a modest inhibition of MuRF1 expression but no modification of MAFbx [292].

#### 4.1.7. NOTCH

The NOTCH pathway is mainly known for its implication in muscle development and regeneration upon injury. However, it has been also implicated in muscle atrophy linked to either cancer or amyotrophic lateral sclerosis (ALS) mice models [161]. Using a tocopherol derivative (AGT251) (Table 2 and Figure 2), the authors found that this antioxidant molecule was protective against muscle atrophy and *MuRF1/Trim63* expression, and that the effects may be mediated through NOTCH1 and 3 expression.

#### 4.1.8. Ion Channels

Electrical stimulation is an important signal that controls muscle mass and ion exchange through specific channels, e.g., K^+^-channels, [296]. Following nerve injury, improvement of muscle mass was observed by blocking K+ channels with 4-aminopyridine (4-AP) [278]. 4-AP (Table 2) was able to partially restore muscle fiber diameter with a concomitant decrease of *MuRF1/Trim63* expression accompanied by decreased *Foxo1* and *Foxo3a* expression.

#### 4.1.9. Acute-Phase Protein Serum Amyloid A1 (SAA1)

Skeletal muscle loss in intensive care unit patients has been at least partially attributed to the acute-phase protein serum amyloid A1 (SAA1) [256] (Table 2). Recent work performed in cultured C2C12 myotubes and septic mice showed that SAA1 effects were mediated through TLR-dependent IL-6 expression and recruitment of the NF-κB pathway. This leads with muscle atrophy and an overactivation of MuRF1/TRIM63, MAFbx/Atrogin-1 and MUSA1 E3 ligases. Using BMS-345541, an inhibitor of the IκB kinase, the authors found that the expression of the E3 ligases returned to basal levels and muscle sparing was observed, indicating that blocking the NF-κB pathway may be an efficient way for indirectly modulating E3 ligases [266].

#### 4.1.10. TGF-β

TGF-β family ligands, including myostatin and activin, are potential effectors of muscle atrophy in several situations of muscle atrophy like cancer [16]. The injection of a truncated form (aa 7-100) of the TGF-β ligands ActRIIB (Table 2 and Figure 2) in mice subjected to several models of cancer cachexia was sufficient for blocking *MuRF1/Trim63* and *MAFbx/Atrogin-1* expression together with complete sparing of both skeletal muscle and heart mass [16].

#### 4.1.11. Reactive oxygen species (ROS)

ROS are downstream modulators of muscle wasting and may be also potential levers for preserving muscle mass [162]. Several molecules have been tested for their potency to modulate E3 ligase expression and thus to preserve muscle mass. Dehydroepiandrosterone (DHEA) (Table 2 and Figure 2), a multifunctional steroid with antioxidant properties was shown to decrease *MuRF1/Trim63* expression (but not *MAFbx/Atrogin-1*) in tumor-bearing rats, which helped moderately preserving muscle mass [168]. Transforming growth factor type beta 1 (TGF-β1) regulates the function and pathological status of skeletal muscle and was found to modulate muscle mass by increasing the activity of NADPH oxidase (NOX), a major ROS producer [69]. This was accompanied by an increased expression of MuRF1. Interestingly, N-acetylcysteine (NAC, a clinically used anti-oxidant) and apocynin (NOX inhibitor) were able to reverse both MuRF1 overexpression and muscle mass loss in cultured myotubes treated with TGF-β1. Similarly, NAC or pyrroloquinoline quinone (PQQ, a naturally occurring antioxidant) were able to decrease *MuRF1/Trim63* and *MAFbx/Atrogin-1* expression and to preserve muscle mass in denervated mice or in starved cultured myotubes [285]. SS-31 is a cell-permeable mitochondria-targeted antioxidant tetrapeptide undergoing clinical trials [297]. This peptide is efficient for lowering ROS production, improving muscle atrophy and decreasing *MuRF1/Trim63* and *MAFbx/Atrogin-1* expression [287]. While ROS modulation seems to be efficient for protecting muscle mass, the mechanisms involved in the decrease of E3 ligases expression is far from being understood. Vitamin E is another antioxidant that has been used in a rat model of muscle disuse (hindlimb suspension) [291]. Vitamin E supplementation was able to largely prevent the overexpression of several proteolytic enzymes including MuRF1/TRIM63 and MAFbx/atrogin-1 but the impact on muscle mass fiber cross section was moderate. Interestingly, the authors attributed the protective role of vitamin E to a direct action on gene expression and not to its antioxidant properties [291].

#### 4.1.12. Leucine and Its Derivative ß-Hydroxy-ß-Methylbutyrate (HMB)

The essential amino acid leucine and its derivative HMB were described as modulators of protein synthesis through an action on the mTORC1 pathway [298,299]. The efficiency of HMB and Leucine on *MuRF1/Trim63* expression was addressed in Dex-treated rats [282] (Table 2). However, while HMB and leucine ameliorated muscle function and decreased MuRF1 expression, no effect of both HMB and leucine was observed on muscle weight. This might be due to partial effect of the treatment on muscles. Interestingly, the modulation of FOXO1 nuclear translocation was the putative mechanism for *MuRF1/Trim63* downregulation. Leucine was also implicated in the modulation of both MuRF1/TRIM63 and MAFbx/atrogin-1 with an improvement of myotube diameter in Dex-treated primary muscle cells [283,300]. The authors found that the effect of leucine on E3 ligase expression was mediated by FOXO3a cytoplasmic sequestration and concomitant vacuolar protein sorting 34 (VPS34) nuclear accumulation. Alternatively, a supplementation with Vital01 (composed by high levels of BCAAs, increased ratio of whey and casein proteins, vitamin D, and ursolic acid) in calorically restricted mouse model of muscle atrophy preserved muscle mass both during and after the atrophic conditions were stablished. The catabolic phenotype was ameliorated by Vital01, notably through the modulation of the UPS (decreased expression of *MuRF1/Trim63* and *MAFbx/Atrogin-1*) and the autophagy-lysosome pathways, [301]. However, Leu and HMB exhibit no effect on E3 ligase expression (*MuRF1/Trim63* and *MAFbx/Atrogin-1*) in human during fasting [210] and the beneficial muscle sparing was attributed to a stimulation of the mTORC1 pathway [298]. On the whole, the potential beneficial effect of Leu and HMB is still controversial for both its action on E3 ligases and for muscle preservation effect.

#### 4.1.13. Plant Derivatives

Plant derivatives were also tested for their potency to protect skeletal muscle atrophy. Ursolic acid (Table 2), was able to partially decrease muscle atrophy in mice subjected to chronic kidney disease and a moderate effect on MuRF1/TRIM63, MAFbx/Atrogin-1 and MUSA1 expression was observed, that was attributed to decreased expression of myostatin and inflammatory cytokines [255]. However, ursolic acid was unable to modify E3 ligases expression in cultured myotubes treated with Dex, and ursolic acid was able to directly induce the expression of *MuRF1/Trim63* and *MAFbx/Atrogin-1* in C2C12 myotubes. More investigation is clearly needed before concluding of any potential therapy using ursolic acid. A polyphenol from green tea, epigallocatechin-3-gallate (EGCG), was also used as a countermeasure for fighting against cancer cachexia [279]. EGCG was able to reduce NF-κB expression and the downstream E3 ligases MuRF1/TRIM63 and MAFbx/Atrogin-1 (only a trend for MuRF1/TRIM63). However, the decrease of the tumor volume renders difficult the interpretation of the effect of ECGC as its protective role on muscles might be indirect. Teaghrelin, an analog of the human ghrelin, was efficient for decreasing the catabolic effect of Dex in cultured C2C12 myotubes, with depressed expression of *MuRF1/Trim63* and *MAFbx/Atrogin-1* [289]. The authors suggested that increased myogenin expression might be implicated in the beneficial effect of teaghrelin. In rats submitted to thermal injury, ghrelin blunted the expression of *MuRF1/Trim63* and *MAFbx/Atrogin-1* [302]. While the exact mechanism was not addressed, the authors found that TNFα and IL-6 mRNA levels were normalized upon ghrelin infusion. Interestingly, mice knocked out for ghrelin exhibit an increased expression of *MuRF1/Trim63* and are less protected from fasting atrophy [290]. Sabinene is a terpene present in plant essential oil and was found to decrease muscle atrophy in starved rats through reversal of the increased *MuRF1/Trim63* overexpression that is commonly observed upon fasting [286]. The mechanism proposed by the authors was the repression of ROS-mediated activation of ERK and p38 MAKP.

Matrine (Table 2 and Figure 2) is a natural compound used in traditional medicine and approved for cancer therapy in China [284]. The authors demonstrated that this compound was able to partially reverse muscle atrophy in mice subjected to Colon 26 adenocarcinoma with a concomitant decrease of *MuRF1/Trim63* and *MAFbx/Atrogin-1* expression. Using cultured C2C12 myotubes, the authors found that the effect of matrine was mainly driven by the AKT/mTORC1/FOXO3a signaling pathway with both a repression of the catalytic axis and an up regulation of the anabolic one.

### 4.2. E3 Ligases Inhibitors

The main E3 ligase that has been investigated so far for the design of inhibitors is MuRF1/TRIM63. This can be explained by the fact it is also the only E3 ligase known to target contractile proteins from both the thin and the thick filament [227,228,230,303].

In a first attempt, the screening of a small molecule library for finding MuRF1/TRIM63 inhibitors identified a compound (P013222) (Table 2 and Figure 2) that was able to decrease MuRF1/TRIM63 autoubiquitylation [294]. The selectivity was within the µM range with a 10 times preference for MuRF1/TRIM63 compared to other E3 ligases and P013222 was able to inhibit the degradation of MHC in Dex-treated C2C12 myotubes.

More recently, the screening of a library identified another small molecule compound (ID#704946/MyoMed-946) able to alter MuRF1-titin interaction (IC50 around 25 µM), thus targeting the coiled-coil region of MuRF1/TRIM63 [293]. Compound ID#704946/MyoMed-946 was able to decrease in vitro MuRF1/TRIM63 self-ubiquitination and surprisingly was also able to decrease the mRNA levels of *MuRF1/Trim63* in catabolic C2C12 myotubes [293]. This suggests that this compound may be interfering on several mechanisms modulating MuRF1/TRIM63 action. This compound was at least partially effective for preserving muscle mass in catabolic mice. The mechanism by which compound ID#704946/MyoMed-946 preserve muscle function needs further investigations as the same laboratory found that it was also able to modulate MuRF2 expression [17,18].

The cellular inhibitor of apoptosis 1 (cIAP1) E3 ligase is a negative regulator of muscle mass by acting on TNFα-mediated NF-κB signaling. cIAP1 is in fact an E3 ligase whose role is to blunt the non-canonical NF-κB signaling and its genetical ablation was reported to improve muscle mass in *mdx* mice [91]. Recently, an inhibitor of cIAP1 (LCL161) was addressed for its capacity to improve skeletal muscle mass in denervated mice [19]. While genetic ablation of cIAP1 was able to preserve muscle mass in denervated mice, its inhibition by LC161 was only moderately efficient as only the EDL muscle was preserved, indicating either a poor inhibition efficiency of LCL161 or a compensation by other E3 ligases and/or signaling pathways.

CBL-B is an E3 ligase involved in the targeting of the Insulin Receptor Substrate 1 (IRS1) that mediates IGF1 signaling, notably by activating the AKT-mTORC1 pathway. CBL-B is involved in spaceflight-induced muscle atrophy and genetic ablation of CBL-B protects skeletal muscle from disuse atrophy [171]. CBL-B can be inhibited by a small pentapeptide mimetic of tyrosine608-phosphorylated IRS-1 that restores IGF1 signaling and protects from atrophy. Interestingly, IGF1 signaling restoration induced a concomitant decrease of MAFbx expression while no variation on *MuRF1/Trim63* mRNA levels was observed [171]. Another peptide, called cblin, was also reported to exhibit some protective action on skeletal muscle through the inhibition of Cbl-b [304].

## 5. Conclusions and Future Directions

The discovery of molecules able to lower muscle loss during catabolic situations is a promising field of investigation and numerous possibilities can be envisaged, from directly blunting the signals arriving at the cellular membrane levels to more specifically inhibiting the E3 ligase(s) involved in the degradation of the muscle contractile apparatus. Each strategy has advantages and disadvantages. The first approaches are not specific and alter numerous metabolic pathways, which may end up with side effects both at the short- and long-term levels. For example, suppressing the general protein breakdown by acting on the PI3K/AKT/FOXO pathway might be deleterious by accumulating misfolded proteins. On the other side, receptor or metabolic pathways have been studied for decades and several inhibitors have been well characterized, which allows more straightforward investigations dedicated to muscle atrophy.

The drugs targeting directly the E3 ligases, so far mostly focused on MuRF1/TRIM63, have the advantage of being more selective and should prove to be better tolerated by the muscle cells and the whole organism. Indeed, MuRF1/TRIM63 (and some other ligases) is muscle-specific, which means that drugs will only affect muscles. This is an important advantage over metabolic pathways that are shared by several organs. More investigations are clearly needed for ameliorating the first generation of molecules or for finding new ones, which includes new strategies for modulating E3 ligases activity.

## Figures and Tables

**Table 1:** Phenotypes of transgenic mice for genes encoding ubiquitin ligases involved in the control of muscle mass and function.

Gene Product	E3 Family	Mouse Model	Phenotype	References
**E3 ligases regulating the anabolic pathways**				
CBL-B	RING	KO	Protection from unloading-induced muscle atrophy and dysfunction	[171]
FBXO40	RING	KD	Myofibers hypertrophy	[22]
		KO	Muscle hypertrophy	
NEDD4-1	HECT	OX	Myocardial activation of AKT during I/R	[176,177]
		KO	Partially resistant to denervation-induced skeletal muscle atrophy	[178]
**E3 ligases regulating the catabolic pathways**				
TRAF6	RING	m.KO	Resistance to starvation induced muscle atrophy	[179]
		m.KO	Resistance to denervation-induced loss of muscle mass and function	[180]
cIAP1	RING	KO	Limitation of denervation-induced muscle atrophy	[19]
		OX	Myotube atrophy	
WWP1	HECT	KD	Muscle fiber atrophy	[181]
TRIM32	RING	KO	Muscular dystrophy	[182]
		DN	Muscular dystrophy	[183]
**Other E3 ligases involved in the control of muscle mass and function**				
MuRF1	RING	KO	Resistance to catabolic-induced muscle atrophy	[4]
MAFbx	RING	KO	Resistance to catabolic-induced muscle atrophy	[4]
PARKIN	RBR	KO	Impaired mitochondrial function and muscle atrophy	[184]
		OX	Increased muscle mass and function in young and old mice	[185]
		OX	Prevention of sepsis-induced muscle atrophy	[186]
SMART/FBXO21	RING	KD	Resistance to denervation-induced muscle atrophy	[187]
MUSA1/FBXO30	RING	KD	Resistance to denervation-induced muscle atrophy	[77]
FBXL21	RING	HM	Impaired muscle functions	[188]
UBR4	HECT	KD	Muscle hypertrophy	[189]
UBR5	HECT	KD	Muscle atrophy	[190]

DN, Dominant Negative mutation; HM, Hypomorphic Mutation; I/R, Ischemia/Reperfusion; KD, knock-down mutant; KO, Knock-out mutant; m.KO, skeletal muscle–specific KO mice; OX, overexpressing mutant; PTEN, Phosphatase and tensin homologue.

**Table 2:** Treatments influencing E3 ligases expression and/or activity.

E3 Ligases Inhibited	Molecule	Mode of Inhibition	Signal inhibited/Activated	Efficiency on E3 Ligases	Efficiency on Muscle Mass	References
**Indirect inhibition of E3 ligases**						
MuRF1/MAFbx Expression	4-aminopyridine (4-AP)	K+-channels blockade	K+-channels blocking	Yes	Yes	[278]
MuRF1 Expression	AGT251	*Notch1*, *Notch3* expression inhibition	NOTCH	Yes	Yes	[161]
MuRF1/MAFbx/MuSA1 Expression	Anti-TLR2	IKK2 (NF-κB)	TLRs Serum Amyloib A1	Yes	Yes	[256]
MuRF1 Expression	Anti-TLR4	IKK2 (NF-κB)	TLRs Serum Amyloib A1	Yes	Yes	[256]
MuRF1/MAFbx/MuSA1 Expression	BMS-345541	IKK2 (NF-κB)	TLRs Serum Amyloib A1	Yes	Yes	[256]
MuRF1 expression	C188-9	STAT3 inhibition	STAT3 signaling	ND	Partially	[14]
MuRF1/MAFbx Expression	Clenbuterol	AKT-FOXO axis	Activation of PI3K-AKT	Yes	Yes	[15]
MuRF1 not MAFbx	Dehydroepiandrosterone (DHEA)	ND	ND	Yes	Yes	[168]
MuRF1 Expression	Epigallocatechin-3-gallate/EGCG	ND	NF-κB	Yes	Yes	[279]
MuRF1 Expression	Espindolol	ND	Myostatin and NF-κB	Yes	Yes	[280]
MuRF1/MAFbx Expression	Formoterol	ND	ND	Yes	Yes	[276]
MuRF1/MAFbx Expression	Formoterol	AKT/mTORC1/FOXO1	ß2 Adrenergic receptor?	Yes	Yes	[277]
MuRF1/MAFbx Expression	Formoterol	ND	AKT and NF-κB	Yes	Yes	[281]
MuRF1/MAFbx Expression	HMB	IL-6 receptor inhibition	NF-κB	Yes	Partially	[275]
MuRF1 expression	HMB or Leucine	FOXO1 nuclear translocation	Glucocorticoid	Yes	No	[282]
Cbl-b activity	IRS1 peptide mimetic	Cbl-b targeting	Activation of PI3K-AKT	Yes	Yes	[171]
MuRF1/MAFbx Expression	Leucine	ND	FOXO3a and VPS34 nuclear translocation	Yes	Yes, myotube diameter	[283]
MuRF1/MAFbx Expression	Matrine	AKT/mTORC1/FOXO3α	FOXO3a and VPS34 nuclear translocation	Yes	Yes	[284]
MuRF1 expression	MR16-1	Anti-IL-6 receptor	NF-κB	Mitigated	No	[275]
MuRF1 expression	N-acetyl cysteine	ROS	TGF-ß	Yes	Yes	[69]
MuRF1/MAFbx Expression	Pyrroloquinoline quinone (PQQ)	ROS	ND	Yes	Yes	[285]
MuRF1/MAFbx Expression	RU486	GR	Glucocorticoid	Yes	ND	[13]
MuRF1 Expression	Sabinene	ROS	ERK, p38 MAPK	Yes	Yes	[286]
MuRF1/MAFbx Expression	sActRIIB	ActRIIB antagonist	SMADs	Yes	Yes	[16]
MuRF1/MAFbx/MuSA1 Expression	Salicylate	IKK2 (NF-κB)	NF-κB	Yes	Yes but toxic	[89]
MuRF1/MAFbx Expression	SS-31	ROS	No	Yes	Yes	[287]
MuRF1/MAFbx Expression	Teaghrelin	ND	Myogenin	Yes	Moderate	[288,289,290]
MuRF1/MAFbx Expression	TNF-BP	TNF binding	TNF	Yes	ND	[13]
MuRF1/MAFbx/MuSA1 Expression	Ursolic acid	ND	Myostatin and inflammatory cytokines	Yes	Moderate	[255]
MuRF1/MAFbx Expression	Vitamin E	ND but seems ROS independent	Unknown	Yes	Moderate	[291]
MuRF1/MAFbx Expression	Vitamin-D	IL-6 receptor inhibition	NF-κB	Yes	Partially	[275]
MuRF1 expression	VX-745/Neflamapimod	p38α MAPK	p38α MAPK	Partially	Moderate	[292]
**Direct inhibition of E3 ligases**						
MuRF1 expression	ID#704946/MyoMed-946	ND	MuRF1 Expression	Yes	Partially	[293]
MuRF1 Expression	ID#704946/MyoMed-946	ND	MuRF1 and MuRF2 Expression	Yes	Partially	[17,18]
MuRF1 and MuRF2 Expression	MyoMed-205	ND	MuRF1 expression			[17]
MuRF1 activity	P013222	MuRF1 targeting	--	Yes	ND	[294]
cIAP1 (*activity??)*	LCL161	cIAP1	NF-κB	Yes	Very moderate	[19]

## Data Availability

No new data were created or analyzed in this study. Data sharing is not applicable to this article.

## Abbreviations

AMPK	Adenosine 5′-monophosphate-activated (AMP)-activated protein kinase
ATG9	Autophagy related gene 9
BMP	Bone Morphogenic Protein
CaMKKß	Ca^2+^/calmodulin-dependent protein kinase kinase ß
cAMP	cyclic Adenosine Monophosphate
CHF	Congestive Heart Failure
CKD	Chronic Kidney Disease
Cn	Calcineurin
CSL	CBF1, Suppressor of Hairless, Lag-1
DMD	Duchenne Muscle Dystrophy
Dsh	Dishevelled
EDL	Extensor digitorum longus
ERK	Extracellular signal-regulated kinases
Fd	Frizzled
FOXO	Forkhead box protein O
GC	Glucocorticoids
GR	Glucocorticoids Receptor
GDF	Growth Differentiation Factor
GPCR	G-protein coupled receptors
HBM	ß-hydroxy-ß-methylbutyrate
HDAC4	Histone deacetylase 4
HECT	Homologous to E6-Associated Protein C Terminus
IGF1	Insulin-like growth factor 1
IKK	IκB Kinase
KD	Knock Down
KO	Knock-Out
LKB1	Liver kinase B1
MAFbx/Atrogin-1	Muscle atrophy F-box
MAPK	Mitogen Activated Protein Kinase
Mdx	The mdx mouse has a point mutation in its DMD gene (coding for Dystrophin)
MSTN	Myostatin
mTORC	Mechanistic (or mammalian) target of rapamycin complex
MuRF1	Muscle Ring-Finger 1 Protein
MyoD	Myogenic regulatory factor
NAC	*N*-acetyl cysteine
NFAT	Nuclear factor of activated T-cells
NICD	Notch Intracellular Domain
NOX	NADPH oxidase
PTEN	Phosphatase and tensin homologue
PDK1	3-phosphoinositide-dependent protein kinase 1
PGC-1a	Peroxisome proliferator-activated receptor gamma coactivator 1-alpha
PI3K	Phosphoinositide 3-kinase
PKA	cAMP-dependent protein kinase
PQQ	pyrroloquinoline quinone
RBR	RING-in-Between-RING
RING	Really Interesting New Gene-finger
RNS	Reactive Nitrogen Species
ROS	Reactive Oxygen Species
SDEN	Surgical sympathetic denervation
SMAD	Small Mothers Against Decapentaplegic
TA	Tibialis Anterior
TAK-1	transforming growth factor ß-activated kinase 1
TAZ/WWTR1	WW domain containing protein 1
TFs	Transcription factors
TGF	Transforming Growth Factor
TRADD	TNF receptor associated via death domain
TRAF6	TNF receptor-associated factor 6
TSC	Tuberous Sclerosis Complex
ULK1	uncoordinated 51-like kinase 1
UPS	Ubiquitin-Proteasome System
Wnt	Wingless-type mouse mammary tumor virus integration site
YAP	Yes-Associated Protein
